# The role of social cognition in decision making

**DOI:** 10.1098/rstb.2008.0156

**Published:** 2008-10-01

**Authors:** Chris D Frith, Tania Singer

**Affiliations:** 1 Center for Functional Integrative Neuroscience, Aarhus University HospitalNørrebrogade 44, Building 30, 8000 Århus C, Denmark; 2 Wellcome Trust Centre for Neuroimaging at University College London12 Queen Square, London WC1N 3BG, UK; 3 Center for Social and Neural Systems, University of ZurichBlümlisalpstrasse 10, 8006 Zurich, Switzerland

**Keywords:** social cognition, mirror neurons, joint action, empathy, theory of mind, trust

## Abstract

Successful decision making in a social setting depends on our ability to understand the intentions, emotions and beliefs of others. The mirror system allows us to understand other people's motor actions and action intentions. ‘Empathy’ allows us to understand and share emotions and sensations with others. ‘Theory of mind’ allows us to understand more abstract concepts such as beliefs or wishes in others. In all these cases, evidence has accumulated that we use the specific neural networks engaged in processing mental states in ourselves to understand the same mental states in others. However, the magnitude of the brain activity in these shared networks is modulated by contextual appraisal of the situation or the other person. An important feature of decision making in a social setting concerns the interaction of reason and emotion. We consider four domains where such interactions occur: our sense of fairness, altruistic punishment, trust and framing effects. In these cases, social motivations and emotions compete with each other, while higher-level control processes modulate the interactions of these low-level biases.

## 1. Introduction

Neuroeconomics, the subject of this special issue, is concerned with the neural basis of decision making. In this contribution, we will review the experiments exploring the role that social cognition plays when individuals make decisions. Neither social cognition nor, indeed, decision making are unitary cognitive processes. So, our review will cover a number of different themes, rather than presenting a unified framework. However, one theme that emerges as somewhat more all encompassing than others concerns the tension between reason and emotion when making decisions. This may overlap with the conflict between automatic and controlled processes. This tension particularly arises in the social domain. We shall briefly explore this theme in the last part of this paper.

A recent definition of social cognition is as follows: ‘Social cognition explains the mechanisms of social behavior using concepts and methods shared with related fields of cognitive psychology and cognitive science’ (Winkielman & Schooler in press). The study of social cognition has a long history, but this study has been revitalized in recent years by the advent of non-invasive brain imaging techniques leading to the new discipline of *social cognitive neuroscience* (Ochsner & Lieberman 2001). The advantage of the cognitive approach to psychology in general and to social behaviour in particular is that, by specifying the underlying processes using terms borrowed from information theory and computer science, it is possible to bridge the gap between mental processes and brain function. Decision making is just one of the many areas in which social cognition has a role. In this contribution, we will only discuss decision making by individuals. We will not discuss the fascinating topic of how social groups make decisions, since, as yet, there are few experiments attempting to link group decisions with neural processes.

## 2. The brain's mirror systems

The discovery of mirror neurons in the frontal and parietal cortex of the monkey brain (Rizzolatti & Craighero 2004) has had a huge impact on social cognitive neuroscience. Mirror neurons become active, not only when the monkey makes a specific action (e.g. picking up a peanut), but also when the monkey observes someone else making the same action. While claims that the existence of mirror neurons can explain social cognition in general, ranging from imitation to language development and theory of mind, are certainly exaggerated (e.g. Jacob & Jeannerod 2005), mirror systems are relevant to some fundamental processes in social interaction. Through the automatic activation of mirror systems when observing the movements of others, we tend to become aligned with them in terms of goals and actions.

In humans, it is only rarely possible to measure the activity in single neurons (but see Hutchison *et al*. 1999), so a slightly different definition of a mirror system is applicable to data from brain imaging. A brain region is considered to be part of a mirror system or a shared network if it is activated during performance of the action as well as during the observation the same action being performed by another person. Of course, this effect might occur even if there were no mirror neurons present in that region, but rather intermingled populations of action and observation neurons (Dinstein *et al*. 2007). Using this definition, shared networks have been identified for sensations, emotions as well as for motor actions. For example, activity can be elicited in primary and secondary somatosensory regions, not only when the subject is touched, but also when the subject sees someone else being touched in the same place (Keysers *et al*. 2004; Blakemore *et al*. 2005). The extension of the idea of mirror systems to the domain of emotions has led to empathy research in the context of social neuroscience (see below).

### (a) Imitating action

The mirror effects that occur when observing motor actions can occur without awareness and seem to be largely involuntary. Observing someone performing an action different from our own, for example moving their arm up and down while we are moving ours from side to side, will cause our actions to become more variable and less accurate (Kilner *et al*. 2003). In this experiment, the effect was specific to observation of people. It did not occur when the movements were being made by a robot arm. A more recent experiment (Stanley *et al*. 2007) showed that this effect depends on who the subjects think the movements are being made by. In the experiment by Stanley and colleagues, a dot was presented, which moved with either a biologically plausible or implausible velocity profile. However, the interference was determined, not by the velocity profile, but by whether the subjects thought the dot represented human- or computer-generated movement. Thus, our mirroring of the actions of others seems to depend upon whether or not we believe them to be people like ourselves, with whom we can engage in social interactions. And indeed this tendency to imitate is at its strongest during social interaction. If the person performing actions is making eye contact with us, strong neural activation is elicited by the movement. But if the person acting has their back to us, their movements elicit very little activity (Kilner *et al*. 2006).

This neural activity elicited by action observation, which is most marked when we are in social contact with another agent like ourselves, can spill over into overt imitation. When two people interact they tend to unconsciously mimic each other's postures, mannerisms and facial expressions (the chameleon effect; Chartrand & Bargh 1999). Furthermore, when this mimicry occurs the interaction occurs more smoothly and the partners like each other better. People with high ratings on empathy scales exhibit this mimicry to a greater extent than others. However, these effects seem to occur only when people are unaware that mimicry is occurring (Lakin & Chartrand 2003).

The experience of mimicry goes beyond an increase in liking for the person who is mimicking us. There is generalized increase in prosocial behaviour. After having been mimicked, we are more likely to donate money to charity even when asked by people who were not directly involved in the mimicry situation (van Baaren *et al*. 2004). Presumably, this is an example of affiliative behaviour causing us to put greater emphasis on the benefits of the group rather than the self when making decisions.

### (b) Joint action

In the case of the chameleon effect, the two people who are interacting generate joint actions in which simple movements are mirrored. However, this imitation (or sharing) can also occur at more abstract levels concerned with goals. This kind of sharing can affect the speed with which decisions are made. Sebanz *et al*. (2003) had subjects perform a choice reaction time task incorporating a spatial compatibility effect. The cue to respond was a finger wearing a red or a green ring, the colour indicating which button should be pressed. However, the finger also pointed left or right. This spatial cue was irrelevant. In one condition, the task was performed by a single subject who simply had to press the left button whenever the ring was red. In this condition, there was no interference from the irrelevant spatial cue, i.e. the reaction time was not slowed when the finger pointed to the right. In another condition, the first subject was joined by a second subject who pressed the right button whenever the ring was green. In this condition, the subject was performing exactly the same task as in the first condition. Nevertheless, a spatial compatibility effect now appeared in the reaction times of both subjects (see also Tsai *et al*. 2006). It seems that when acting together each subject automatically represented the task requirements and goals of the other subject as well as their own. In this particular case, such representations resulted in a slowing of reaction times. An fMRI study of this task (Sebanz *et al*. 2007) showed that performance in the presence of a co-actor elicited increased activity in ventral premotor and orbitofrontal cortices. These results suggest that knowing about the potential actions of a partner increases the relevance of stimuli referring the self and also increases the need to monitor one's actions.

In this example, although the subjects were performing their tasks at the same time, no cooperation was needed to perform these tasks. When joint action requires cooperation, shared representations of task requirements and goals are very important in order to achieve better performance. Such sharing is referred to as *common knowledge* (Clark 1996) or *alignment* (Pickering & Garrod 2004) and has been studied extensively in experiments on discourse. Some of these shared representations are established through cultural norms, but alignment of representations at many levels will develop during the course of an interaction. Once again such alignment largely occurs automatically and with little awareness. Furthermore, this automatic emergence of shared representations occurs only when we interact with biological agents. Again, the interference effect observed by Sebanz and colleagues occurred for a human hand only, but not for a wooden hand (Tsai & Brass 2007).

## 3. Empathy: sharing emotions

Our ability to understand other people is not restricted to our capacity to understand their action intentions. Successful social interaction very frequently also requires an understanding of the other's emotional and motivational states, i.e. our capacity to empathize with others (for a detailed definition of terms, see also de Vignemont & Singer 2006). Our ability to share the emotions of others has long been recognized.

How selfish soever man may be supposed, there are evidently some principles in his nature, which interest him in the fortune of others, and render their happiness necessary to him, though he derives nothing from it except the pleasure of seeing it. Of this kind is pity or compassion, the emotion which we feel for the misery of others, when we either see it, or are made to conceive it in a very lively manner. That we often derive sorrow from the sorrow of others, is a matter of fact too obvious to require any instances to prove it; for this sentiment, like all the other original passions of human nature, is by no means confined to the virtuous and humane, though they perhaps may feel it with the most exquisite sensibility. The greatest ruffian, the most hardened violator of the laws of society, is not altogether without it.

Introduction to *The Theory of Moral Sentiments*, Adam Smith (1759)

The idea that perception–action links in our brains enable us to understand other peoples motor action has been expanded to include the ability to share not only motor actions but also feelings and sensations with others (Preston & de Waal 2002; Gallese 2003; Decety & Jackson 2004; Decety & Lamm 2006; de Vignemont & Singer 2006). Recent empathy studies have indeed brought accumulating evidence for such shared neural networks in the domain of emotions.

Early studies focused on emotional contagion triggered by the mere perception of emotional expressions in faces. It could be observed that the sight of the facial expression of disgust elicits activity in the same regions as direct exposure to a disgusting smell (Wicker *et al*. 2003). Likewise, the sight of a fearful face elicits activity in the same regions as direct exposure to a fearful object (Morris *et al*. 1996). This emotional response to the fear and disgust of others has obvious advantages for survival. If someone looks afraid, then we should be vigilant since there is probably something nearby that we too should avoid. If someone looks disgusted, it is probably wise to check what we are eating ourselves in case the food is bad. However, imitation is not always the most appropriate response to the emotions of others. For example, rather than expressing anger oneself, the most appropriate response to another's expression of anger might well be a complementary expression that achieves appeasement, such as the expression of embarrassment (Keltner & Buswell 1997).

Such shared networks are not only observed when we are presented with emotional stimuli. We also empathize with others when we know that someone is suffering in the absence of any explicit emotional stimulation. Singer *et al*. (2004*b*), for example, measured empathic brain responses in pain-relevant brain areas elicited by the sheer knowledge that your partner suffered pain. More specifically, she recruited couples and measured empathy *in vivo* by assessing brain activity in the female partner while painful stimulation was applied either to her own or to her partner's right hand who was sitting next to her in the MRI room but whom she could not see. Differently, coloured flashes of light on a visible screen pointed to either the scanned subject or her partner's hand, indicating which of them would receive painful stimulation and which would receive non-painful stimulation. This procedure enabled the measurement of pain-related brain activation when pain was applied to the scanned subject (felt pain) or to her partner (empathy for pain). The results suggest that parts of the so-called ‘pain matrix’, predominantly bilateral anterior insula (AI) and the anterior cingulate cortex, were activated when subjects experienced pain themselves as well as when they saw a signal indicating that a loved one had experienced pain. Activation in this network was also observed when subjects saw an unknown but likeable person suffering pain (Singer *et al*. 2006), when subjects watched videos showing body parts in potentially painful situations (Jackson *et al*. 2006), painful facial expressions (Lamm *et al*. 2007) or hands being pricked by needles (Morrison *et al*. 2004, 2007). For a review, see de Vignemont & Singer (2006).

Further studies suggest that empathic brain responses are not just an automatic all or none response, but that the presence and the magnitude of these empathic brain responses can be modulated by different factors such as the affective link to the other person, the perceived fairness of the other (Singer *et al*. 2004*a*, 2006), the subject's appraisal of whether the reason the other person is suffering is justified (Lamm *et al*. 2007), the frequency of a person's prior exposure to pain-inducing situations (Cheng *et al*. 2007) and the intensity of the inflicted pain (seeing a needle pricking versus penetrating a muscle; Avenanti *et al*. 2006). As with the observation of actions, observation of other people's emotions often spills over into overt mimicry. We wince when we see another person in pain. Furthermore, this response is much greater when we are in some sort of social contact with the person we are observing. Bavelas and colleagues (Bavelas *et al*. 1986) measured mimicry when subjects observed the victim of an apparently painful injury. This mimicry was significantly enhanced when the observer and the victim were in eye contact. It seems that, when we believe we are engaged in a social interaction, we experience, or at least express, more empathy.

So far, social neuroscientists have mostly focused on phenomena such as emotional contagion and empathy, whereby the former connotes a reaction in which one shares an emotion with another person without realizing that the other person's emotion was the trigger. By contrast, empathy requires the awareness that our affective state was elicited by another person's affective state. However, how empathy enables the development of other-regarding motivation (empathic concern or compassion) with an associated helping behaviour is still unclear (see also de Vignemont & Singer 2006). Future research will have to focus on the link between empathic brain responses, compassion and behaviour to achieve a better understanding of how empathy and prosocial decision making are linked.

## 4. ‘Theory of mind’ in social interactions

Most social interaction is also strongly influenced by our more abstract beliefs about who we are interacting with rather than the actual behaviour or motivational state of the other. When we are interacting with another person, we assume that they have minds like our own and try to predict their behaviour on the basis of the contents of their minds: their beliefs and desires. This is referred to as having a ‘theory of mind’ (Premack & Woodruff 1978), taking an intentional stance (Dennett 1987), or mentalizing (Frith 1989). Mentalizing has been studied using a wide range of tasks including reading stories (Fletcher *et al*. 1995; Saxe & Kanwisher 2003), looking at cartoons (Brunet *et al*. 2000; Gallagher *et al*. 2000) or watching simple animations (Castelli *et al*. 2000). In spite of the wide range of material used, a small set of brain regions is reliably activated in all these studies including medial prefrontal cortex/paracingulate cortex (MPFC), posterior superior temporal cortex and the temporal poles (see Frith & Frith 2003 for a review). However, a disadvantage of all these studies is that the tasks used are ‘off-line’. That is, the subjects in the studies are not themselves directly engaged in a social interaction. Rather, they are observing and interpreting the interactions of others. By contrast, when the subjects play simple economic games, developed in the framework of game theory, they are directly involved in a social interaction. Mentalizing is important for these economic games also.

### (a) The intentional stance

The importance of who we believe we are interacting with is shown in a series of studies of simple interactive games. In the study of McCabe *et al*. (2001), the subjects played a trust and reciprocity game (iterated Prisoner's Dilemma) against a human counterpart or a computer. In the subjects who cooperated there was more activity in MPFC when playing with a person than with a computer. As we have seen, MPFC is one of the brain regions consistently activated when subjects perform tasks in which they have to think about the mental states of others. Rilling *et al*. (2004) made a similar observation with subjects who played either the ultimatum game (see below) or Prisoner's Dilemma game. Once again, the activity in brain regions concerned with theory of mind, including MPFC, was stronger when playing with a human partner than a computer. The subjects in this experiment were told they were either playing against a human or a computer that chose its responses randomly. In fact, however, whether the partner was said to be human or computer, the play was identical. Thus, the effect was created by what the subjects believed about their partners rather than the actual run of the play.

The same design was used by Gallagher *et al*. (2002) in an experiment where subjects played the game rock–paper–scissors. Greater activity was seen in MPFC when subjects believed they were playing against a human as opposed to a computer. In this case, the computer was said to use simple, predetermined rules based on the subject's last response. Once again, during the critical scanning window, the play of the ‘human’ or the ‘computer’ did not actually differ, being a random sequence. After scanning, subjects were asked to report on their subjective experience with the different opponents. All subjects reported that the two conditions ‘felt’ distinctly different. The experience of playing the human was described as an interaction with a rational agent with distinct beliefs, intentions and desires. By contrast, the computer was described as behaving according to some prior design. This difference was not simply that the human seemed the more difficult opponent. The subjects felt under much greater time pressure when playing against the computer.

These results suggest that, when subjects believe they are paying against another person, they think about the mental states of this person (mentalizing). This ‘intentional stance’ (Dennett 1987) is adopted to a much lesser extent when playing against a computer. However, the results do not reveal what precise effect this intentional stance has on the strategies of play that are used. By its nature the rock–paper–scissors game does not lend itself to strategic analysis. However, there is some evidence about a possible role for mentalizing in the ultimatum game and the related dictator game. For example, a child's ability to mentalize (i.e. successfully perform theory of mind tasks) was found to be positively related to the likelihood of cooperating in Prisoner's Dilemma games and to making fair offers rather than very small proposals in the ultimatum game (Sally & Hill 2006).

In the ultimatum game, one player (the proposer) is given a sum of money and then must choose how much to offer the other player (the responder) who may accept the offer or refuse it. A refusal means that both parties get nothing. If people act on the basis of rational self-interest, then the responder should accept any offer however small. Otherwise he will get nothing. On this basis, the proposer should offer very little, since small amounts should be accepted by the responder. However, this is not what actually happens. In the ultimatum game, the majority of responders will refuse offers of less than a third of the total (see Camerer & Thaler 1995 for a review). The responders consider that small offers are ‘unfair’, a matter to which we shall return later. If the proposer wants his offer to be accepted, he has to take the responder's view of small offers into account. He has to predict whether or not the responder will consider that his offer is fair. In other words, he must think about the responder's view of his offer, an example of mentalizing (see also Singer & Fehr 2005; McCabe & Singer 2008).

This requirement does not apply to the dictator game. In this game, the proposer grants the second player a proportion of the money which that player is bound to accept. So, the proposer has no need to take account of what the responder thinks about fairness, since the responder is obliged to accept his offer. We would therefore expect lower offers to be made in the dictator game. Consistent with this analysis, offers made in the dictator game are indeed significantly lower than in the ultimatum game. Typically, offers made in the dictator game are about half those made in the ultimatum game (Forsythe *et al*. 1994).

However, the offers made in the dictator game are still substantial, at approximately 20 per cent of the total. Why should the proposer give anything away in this game? Here too mentalizing may have a role. The idea of a rational economic man who is concerned only to maximize his wealth is often traced back to Adam Smith's ‘*Wealth of Nations*’. ‘It is not from the benevolence of the butcher, the brewer, or the baker that we expect our dinner, but from their regard to their own interest.’ However, in his *Theory of Moral Sentiments*, Smith suggested that underlying the drive to acquire wealth is a more fundamental desire to acquire a good reputation.

That we are held in consideration is both ‘the most agreeable hope’ and ‘the most ardent desire of human nature’. No one except the perfect sage and the man depraved to the rank of beasts can remain indifferent to the lure of public recognition. There is no price that we are not ready to pay to acquire it, since ‘men have voluntarily thrown away life to acquire after death a renown which they could no longer enjoy’.

Adam Smith's (1759) *Theory of Moral Sentiments* (from Todorov 1996, p. 6)

Models of cooperation have recently incorporated the possibility of ‘image scoring’ and reputation formation as promoters of cooperation (Nowak & Sigmund 1998*a*,*b*; Wedekind & Milinski 2000). That generosity in the dictator game depends, in part, on the wish for a good reputation in the eyes of others is supported by the observation that smaller offers are made when the proposer has complete anonymity (Hoffman *et al*. 1996). The role of the ‘gift’ in society and the prestige it brings have been studied intensively by anthropologists (Mauss 1924) and extends from traditional potlatch rituals where a leader strengthens group relations and acquires honour by giving away large amounts of goods to modern phenomena such as open source software and Wikipedia to which many people freely donate their time and expertise (Zeitlyn 2003).

Thinking about our reputation requires second-order mentalizing. We have to represent what someone else is thinking about us. We have proposed (Amodio & Frith 2006), although as yet the evidence is not strong, that the anterior rostral MPFC might have a special role in this reflexive representation of what others think about us (see also Saxe 2006). This region is activated when thinking about our own mental states as well as when thinking about the mental states of others (Mitchell *et al*. 2005). Activity in this region has also been observed when subjects make unintended responses suggestive of race prejudice, but only when such responses are being observed by others (Amodio *et al*. 2006).

This reflexive form of mentalizing is especially important in games involving repeated economic exchanges. For cooperation to develop in such games it is important to estimate how much I can trust my partner. But it is equally important for me to persuade my partner to trust me and for me to be able to estimate how well I have succeeded in this endeavour. Perhaps this is the role of MPFC, which, as we have already seen, is often activated during the playing of such games, especially when subjects are cooperating (McCabe *et al*. 2001). Of particular interest in this context is the study from Read Montague's group (Tomlin *et al*. 2006). In this experiment both partners in a trust and reciprocity game were scanned. In each of 10 rounds, one player (the investor) invested an amount of money (investment phase) that was tripled and sent to the other player (the trustee) who decided to repay some fraction of the tripled amount (repayment phase). A striking effect was observed in the cingulate cortex such that a different pattern of activity was observed for the investment phase compared with the repayment phase. In particular, the activity in the most anterior region of the anterior cingulate was much greater when a subject learned what his partner was repaying, than when the subject made his own investment. The authors of this study suggest that these differences in activity reflect a mechanism for distinguish ‘me’ and ‘not me’ when assigning credit for the shared outcome. However, we prefer an alternative account concerned with reputation. It is at the point of repayment that a subject learns to what extent he is trusted by his partner and this is the time at which maximal activity is seen in the anterior MPFC.

The need to mentalize, whether to predict what our partner is going to do next or to manipulate our reputation in his eyes, does not arise if we are playing against a computer (Rilling *et al*. 2004) or a person who is simply following a predetermined sequence of instructions (Singer *et al*. 2004*a*). As we have already seen, when subjects believe they are playing against such partners, significantly less activity is observed in brain regions concerned with mentalizing, including MPFC. There are also behavioural changes consistent with a different approach to the game. When the offer in the ultimatum game is based on the spin of a roulette wheel, much lower offers are accepted (Blount 1995). The same result was observed by Rilling *et al*. (2004) when subjects believed the offer was being made by a computer.

## 5. Reason and emotion

While mentalizing certainly plays a prominent role in economic decision making and social exchange, there is also an important role for emotional responses. This has been revealed by studies of the effect of frontal lobe damage on many aspects of decision making (e.g. Bechara *et al*. 2000). In relation to economic decision making, our sense of fairness, altruistic punishment, trust and framing effects are good examples for domains in which emotions can interfere with rational decisions.

### (a) Our sense of fairness

For example, our ability to mentalize may help to determine what the next moves or intentions of the other players might be, but emotions also play an important role in the assessment of the fairness of the offer. It is this feeling of fairness that we will now consider in more detail. We have suggested that one reason a responder does not behave as a rational economic man should is through a desire for reputation rather than wealth. However, a long-standing idea (see Damasio 1994) is rather that rational decisions are spoiled by emotional responses. For example, low offers in the ultimatum game are considered to be unfair and this feeling of unfairness is usually accompanied by strong emotions and activity in brain regions, such as the AI, associated with feelings (Sanfey *et al*. 2003). Furthermore, the higher the activity in the AI the more likely the offer is to be rejected. The justification for this interpretation in terms of emotion is that the activity in the AI has often been associated with subjective feeling states, such as the subjective unpleasantness of painful stimulation (Craig 2002) or the feeling of disgust (Wicker *et al*. 2003): it reflects how pleasant or unpleasant we feel a situation to be. It is this emotional response that makes people behave in an ‘irrational’ way.

### (b) Altruistic punishment

The implicit assumption is that this emotional response makes the decision less than optimal. However, in a group setting, turning down unfair offers can be seen as a good decision. When the responder turns down an unfair offer in the ultimatum game, he is effectively punishing the proposer who will not get any money as a result of the refusal. This is an example of altruistic punishment since the responder foregoes monetary gain in order to punish the proposer.

Altruistic punishment has been shown to have a vital role in maintaining the cooperation in groups (Gintis 2000; Bowles & Gintis 2002, 2004; Fehr & Gachter 2002; Boyd *et al*. 2003). In common good games involving several players, the group benefits from the investments of individual players. Each time an individual invests, the group as a whole gains while the individual investor loses a little. As long as everyone invests, every one also gains. But in such situations *free riders* will inevitably appear. These players accept the benefit from the investments of others while withholding their own money. The free-riding individuals gain at the expense of the group. Once free riders have appeared in the group, cooperation breaks down. If, however, altruistic punishment is possible, then free riding is reduced and cooperation flourishes (Fehr & Gachter 2002). Here, altruistic punishment is applied even though it brings a material loss to the individual player. But while the individual may lose each time he applies punishment, he benefits in the long run from the increased cooperation occurring in the group. A loss to the individual is converted into a gain for the group.

Gurerk *et al*. (2006) compared two experimental institutions, one of which sanctioned punishment while the other was sanction free. In spite of the initial aversion to the institution with sanctions, after some experience with the sanction-less institution subjects migrated to the other institution. The sanctioning institution became strongly cooperative, while the sanction-free institution became depopulated.

Altruistic punishment clearly has an important role in maintaining cooperation in groups. What is its neural basis? de Quervain *et al*. (2004) measured brain activity while subjects learned about a defector's abuse of trust and determined the punishment. Delivery of altruistic punishment was associated with activity in the dorsal striatum and subjects with greater activity in this region were prepared to incur greater costs in order to punish. From both animal and human studies, the striatum (a component of the basal ganglia) is known to have a major role in associating rewards with actions (Delgado 2007).

Related observations were made by Singer *et al*. (2006). In this study, subjects played a trust game with two other players (confederates of the experimenter) in the course of which they learned that one player was a cooperator (playing fairly) while the other was a defector (playing unfairly). Subsequently, the subjects observed the two players receiving pain. Empathy-related activity was observed in pain-related brain areas (AI and anterior cingulate) when the fair player received pain, but this activity was significantly reduced for the unfair player. In addition, in the male subjects, the knowledge that the unfair player was receiving pain was associated with activity in reward-related regions of the striatum. The magnitude of this activity was correlated with the subjects' expressed desire for revenge.

These results suggest that the ‘emotional’ responses observed in these social interactions have an important role in ensuring that the long-term interests of the group are given greater weight than the short-term interests of the individual. They may ensure that, when we are interacting with other people, we have empathy for those who cooperate and a desire to punish those who defect.

### (c) Trust

Another salient dimension in economic decision making often associated with emotional activation in the brain is trust. How do we know whom to trust and how do we learn to trust?

Studies have shown that when confronted with faces of people never seen before, subjects consistently rate some faces as appearing less trustworthy than others. This ability to recognize trustworthiness in faces is impaired in patients with damage to the amygdala (Adolphs *et al*. 1998). Furthermore, confrontation with such apparently untrustworthy faces elicits activity in the amygdala (Winston *et al*. 2002) in normal volunteers. This activity reflects a negative evaluation. These are people to be avoided. This effect also appears to occur rather automatically. It occurs whether we are explicitly rating the faces for trustworthiness, but is just as strong if we are rating the faces for some other feature that is not relevant to trustworthiness.

Although there is good agreement about the kinds of faces that look untrustworthy, there is no evidence that this attribution has any validity. This is an example of prejudice. On the other hand, we can rapidly learn to recognize people as untrustworthy. Bayliss & Tipper (2006) used eye gaze direction as cues in spatial attention task. The faces of some people reliably gazed in the same direction as the upcoming target (valid cues), while other people consistently gazed in the wrong direction (invalid cues). The subjects in this experiment persisted in following gaze direction even when it was consistently invalid. However, they subsequently rated the people who gave the misleading gaze cues as looking more untrustworthy.

When we interact with other people we acquire direct evidence about how trustworthy they are from their behaviour. Singer *et al*. (2004*a*) used the setting of a sequential trust and reciprocity game (iterated Prisoner's Dilemma) to let subjects learn that some people consistently played fairly (cooperators) while others consistently played unfairly (defectors). After this experience, the subjects rated the faces of the defectors, not only as less likeable, but also as less physically attractive. In parallel with these changes in the perception of the faces, there were increases in neural activity elicited by the presentation of faces of cooperators and defectors in comparison to neutral faces. An important feature of this experiment was that some players were presented as freely choosing their responses, while others were said to be simply following the instructions. The behavioural and neural effects were significantly more marked in response to the players who were presented as intentional agents. For example, the presentation of intentional cooperators elicited more activity in the amygdala, insula and reward-related areas such as striatum and orbitofrontal cortex. These are areas generally associated with emotions, in this case positive emotions. This result confirms the suggestion that social cognitive processes are preferentially engaged when people believe they are interacting with intentional and free agents like themselves.

In the experiment just described, the subjects learned about previously unknown people by interacting with them. But we do not have to interact directly with people to find out something about them. We can also learn about their reputation from others. We are told that X is reliable while Y cannot be trusted. Delgado *et al*. (2005) had their subjects read vivid descriptions of potential trading partners indicating praiseworthy, neutral or suspect moral character. Subsequently, the subjects were scanned while they interacted with these partners in a trust and reciprocity game. Although all their partners behaved identically in the game, the subjects were more likely to make risky investments with the partner previously described as ‘good’. During games of this kind, the activity in the striatum is observed, which differentiates between positive and negative feedbacks (i.e. whether trust is reciprocated or not). This is consistent with the idea that the striatum is part of a neural circuit that guides and adjusts future behaviour on the basis of reward feedback (Delgado 2007). However, in the experiment of Delgado *et al*. (2005), very little striatal activity was seen except when interacting with the neutral partner. It appears that once partners had acquired a reputation, the subjects paid much less attention to their actual behaviour in the game.

In a recent experiment by Kosfeld *et al*. (2005), trust, as measured through a sequential trust and reciprocity game, could be increased by administration of oxytocin, a neuropeptide that plays a key role in social attachment and affiliation behaviour in non-human mammals. Interestingly, the authors could show that oxytocin specifically affects an individual's willingness to trust another person, i.e. to accept social risks arising through interpersonal interactions with an intentional agent who may not reciprocate your trust, but was not due to a general increase in the readiness to bear risks in a lottery game with the same pay-offs.

### (d) The framing effect

The framing effect provides another example in which emotion appears to interfere with a rational decision. In this case, if the identical problem is framed in different ways, the frame affects the decision that is made (Tversky & Kahneman 1981). Consider a situation in which a strategy has to be chosen to deal with the outbreak of a potentially fatal illness. Would you take course of action A which will save 200 people (out of 600), or would you choose the more risky action B? This latter option may save every one, but brings with it the risk that no one will be saved. With this frame (the gain frame), the majority of respondents choose the non-risky course A. The same problem can be framed in a different way. Would you take course of action A which will allow 400 people to die (out of 600), or would you choose the more risky action B. This option may save every one, but brings with it the risk that every one will die? With this frame (the loss frame), the majority of respondents choose the risky course B. The first frame emphasizes the possibility that the risky option may result in more deaths than the safe option. The second frame emphasizes the possibility that the risky option may save more lives than the safe option. In fact, the probabilities are the same in both the cases.

De Martino *et al*. (2006) used fMRI to measure brain activity while subjects made decision about problems that were framed in this way. They observed increased activity in the amygdala in association with the framing effect, i.e. when choosing the safe option in the gain frame and the risky option in the loss frame. They concluded that the framing effect is driven by emotional responses.

But, as was the case with responses to unfair offers in the ultimatum game, even if a decision is influenced by emotional factors, this may not be a bad thing in an interactive setting.

In real-life interactions between people, the way that an utterance is framed is by no means irrelevant. People typically communicate more than that which is explicitly stated in their words. Pragmatics is the study of this communicative aspect of language (Levinson 1983). Sher & McKenzie (2006) studied how subjects interpreted the remark, ‘This glass is half full’ as opposed the apparently equivalent remark, ‘This glass is half empty’. Subjects inferred that these remarks revealed the speakers expectations about the glass. If the speaker had expected the glass to be full then they would say, ‘This glass is half empty’ thereby emphasizing the change. Sher and McKenzie call the implications of the way something is framed as information leakage and suggest that these implications are made by the speaker and drawn by the listener at a largely unconscious level. At this level of processing, frames affect decisions in much the same way that facial expressions or gestures. The information provided by such framing can be useful. However, frames can also be deliberately and consciously manipulated so that messages can be rendered misleading or outright deceptive. This technique is now widely practiced by politicians (Scheufele & Tewksbury 2007).

## 6. Cognitive control in decision making

If emotion is seen as the enemy of reason, then we would expect to find high-level mechanisms for controlling emotion and preventing its damaging effects. This was the interpretation for the study of the framing effect put forward by De Martino *et al*. (2006). Across the subjects in this experiment, the activity in orbital and ventromedial PFC predicted a reduced susceptibility to the framing effect. Similar regions are proposed as sources for the more general control of emotion in the review by Ochsner & Gross (2005). These regions of PFC are seen as enabling us to overcome the undesirable effects of emotion.

### (a) Economic decision making

As we have seen in the case of the ultimatum game, the decisions generated by emotional responses need not *per se* be classified as poor decisions. The emotional response to an unfair offer may be the basis for altruistic punishment, which, in turn, can increase cooperation and hence may provide greater rewards at the level of the group. In this case, it would seem inappropriate for high-level control systems to be brought into play to override the effects of emotions. A more plausible scenario is for there to be competition between two competing tendencies: to benefit the self or to benefit the group (Fehr & Camerer 2007).

To investigate the role of PFC in the control of such motivational response tendencies, Knoch *et al*. (2006) used TMS to disrupt specific regions of the PFC while subjects played the ultimatum game. Application of TMS over right but not left dorsolateral PFC (DLPFC) caused subjects to accept unfair offers even though they still recognized that these offers were unfair. These results suggest that functions of the DLPFC usually help inhibit self-interested tendencies, in this case, to take all the money offered irrespective of whether social norms are violated or not. In contrast to this result is the study by Koenigs & Tranel (2007) of patients with lesions to ventromedial PFC. These patients showed the opposite tendency: they were more likely to reject unfair offers. Does this reflect a failure to inhibit prosocial tendencies?

In general, high-level control comes into play when we try to override rather automatic tendencies. Inhibitory control can be applied as much to prosocial motivation and behaviour as to self-interest. This idea is explored in a recent study by Rilling *et al*. (2007). In this study, brain activity elicited during a trust and reciprocity game was related to psychopathy scores in a sample of students. Both brain activity and behaviour suggested that students with low psychopathy scores had a bias to cooperate, while those with high psychopathy scores had a bias to defect. Of particular interest were those trials where the students acted against type, i.e. low scorers defecting and high scorers cooperating. In both the cases, these trials were associated with greater activity in dorsolateral prefrontal cortex (DLPFC). Rilling and colleagues suggest that this activity in DLPFC reflects the effortful exertion of high-level cognitive control. This is consistent with many accounts of the role of DLPFC as selecting between competing action possibilities (e.g. Frith 2000). We further speculate that such control is possible only when we are aware that these automatic biases are in play.

### (b) Moral dilemmas

There are remarkable parallels between the economic decisions we have discussed above and moral decisions. In both the cases, there seems to be a conflict between reason and emotion and the same brain regions are implicated. The rational approach to moral decisions is often referred to as utilitarianism and has the same roots as the idea in economics that the correct choice in any decision is the one that maximized utility (e.g. wealth or happiness). The principles behind moral choices can be revealed by presenting subjects with moral dilemmas, in which a choice has to be made between two bad outcomes. A famous example is known as the trolley problem.

A trolley is running out of control down a track. In its path are 5 people who have been tied to the track by a mad philosopher. Fortunately, you can flip a switch which will lead the trolley down a different track to safety. Unfortunately, there is a single person tied to that track. Should you flip the switch or do nothing?

The ‘rational’ utilitarian answer is that you should flip the switch since five deaths are worse than one death and most people agree that it would be right to flip the switch (Greene *et al*. 2001). However, with another version of the dilemma the opposite result is obtained.

A trolley is hurtling down a track towards five people. You are on a bridge under which it will pass, and you can stop it by dropping a heavy weight in front of it. As it happens, there is a very fat man next to you—your only way to stop the trolley is to push him over the bridge and onto the track, killing him to save five. Should you proceed?

Confronted with this dilemma most people say no. From a utilitarian point of view, this problem is no different from the previous one. So why do people typically make a different decision? Greene and colleagues confronted subjects with these problems while they were being scanned and report that the second trolley problem elicits greater activity in brain regions concerned with emotional processing. So, the argument is that the emotional response to the thought of directly killing someone by throwing them into the path of the trolley interferes with the rational (i.e. utilitarian) processes of decision making when confronted with moral dilemmas. This is the same explanation as for the irrational behaviour in the ultimatum game.

### (c) The effect of frontal lobe damage on social decisions

The implication here is that in the absence of an emotional response, we would choose the more utilitarian option. This prediction was examined (Koenigs *et al*. 2007) by studying the patients with lesions to ventromedial prefrontal cortex (VMPC), since such patients have reduced emotional responses. The prediction was confirmed. These patients chose the utilitarian option even though such options involved emotionally aversive behaviours such as pushing a fat man into the path of the trolley.

Note, however, that this is the opposite result to that obtained with the same patients when they played the ultimatum game (Koenigs & Tranel 2007). In this case, the patients, as we have seen, were more likely than comparison groups to reject unfair offers, i.e. they rejected the utilitarian option due to an exaggerated response to unfair offers. Given these contradictory results, a simple explanation in terms of altered emotional responsiveness does not seem viable. There is, however, a critical difference between studies of moral dilemmas and studies of economic decision making. In the neuroeconomic studies, the subjects were actually making decisions and gaining or losing money. By contrast, the studies of moral dilemmas were *off-line*. No decisions were made and there were no consequences. Rather, subjects indicated what decisions *ought* to be made. We do not know (fortunately) what decisions they would have made if confronted with such dilemmas in real life. So, the experiments on moral dilemmas are not really about decisions, but about knowledge of social rules.

A striking feature of patients’ VMPC damage is that they can have marked abnormalities of decision making, especially in relation to social conduct, while having preserved conscious knowledge of the appropriate behaviour in social situations (e.g. Saver & Damasio 1991). The problem for these patients is that their frontal lobe damage prevents them from using this knowledge to override the various low-level biases, discussed above, that influence our decision making. This can result in inappropriate cooperation (e.g. entering into ill-advised business partnerships; Eslinger & Damasio 1985) as well as inappropriate selfishness. Perhaps this dissociation between high-level conscious knowledge and low-level biases produces abnormalities in the other direction as well. When consciously accessing the rules of social behaviour, rather than actually making decisions, patients with VMPC damage may be less influenced by low-level biases elicited by the frame in which the decision scenario is presented. As a result, their responses are more utilitarian.

## 7. Conclusions

In the previous section, we have emphasized different aspects of social cognition which influence decision making: emotional intuitions, motivational bias and high-level executive control processes. For example, there is a bias to be prosocial and to maximize gains for the group, and this motivational bias competes with another bias to maximize gains for the self. This competition can also be solved by high-level control mechanisms. These control mechanisms can, to some extent, override emotional impulses and motivational biases of either kind. This high-level control is also strongly social in character. We are taught to recognize our low-level biases and overcome them. It is at this level that culture and social norms have their effect (Shweder *et al*. 1990). We know very little about how these high-level social effects operate at the neural level. We have, however, summarized some evidence that lower-level responses can be strongly modulated by whether we believe another person is an intentional agent or not and thus responsible for its actions as well as whether we belief this person's actions were fair or justified. Furthermore, hints for cultural influences are beginning to emerge from various sources including studies of race prejudice.

As Phelps & Thomas (2003) remind us, 40 years ago it was not uncommon for white Americans to express negative attitudes to black Americans. However, recent studies have shown that white Americans’ explicit attitudes are significantly less biased today. This is presumably a cultural effect. But at the same time there is robust evidence that, when attitudes are assessed implicitly, most white Americans still demonstrate a negative attitude towards black Americans (e.g. Phelps *et al*. 2000). The greater this implicit (unconscious) prejudice, the greater the response in the amygdala elicited by the presentation of the faces of unknown black Americans. However, the magnitude of this activity is not correlated with explicit measures of prejudice. This is clear evidence for the independence of implicit and explicit attitudes at the neural level. In a subsequent experiment (Cunningham *et al*. 2004), the faces of the black Americans were presented, either very briefly (30 ms) or for a longer period (535 ms). The amygdala activation associated with implicit (unconscious) race prejudice was much reduced when the faces were presented for the longer period. Furthermore, the magnitude of activity in PFC predicted how much the amygdala activity would be reduced for the long presentations. Cunningham *et al.* concluded that this is evidence that activation in dorsolateral PFC and anterior cingulate is associated with attempts to control unwanted prejudicial responses to black faces. A key question for future neuroimaging research will be to investigate whether it is necessary to become aware of these automatic prejudices for the high-level control processes to come into play (see Tsushima *et al*. 2006 for evidence for this idea outwith the realm of prejudice).

These same processes apply to economic and moral decisions also. On the one hand, our decisions are guided by fast and largely unconscious intuitions as to what feels right. On the other hand, there are conscious, deliberate and rationalized (rather than rational) processes strongly influenced by education and culture (Loewenstein 2000; Engel & Singer 2008). This is essentially the dual-process model of reasoning described by Evans (2003). These two social processes are seen most starkly in studies of law, which is, indeed, a mechanism for making decisions in the context of moral dilemmas. In Anglo-American jurisprudence, the distinction is made between an intuition-based sense of justice and reason-based dictates of law (Goodenough & Prehn 2004). It has long been recognized that both these two systems are important for the development of the law. For example, considerations of natural justice are required in order to declare that some specific law is unjust and should be changed. The studies of economic decision making we have reviewed here suggest that our intuitive ‘emotion’-based decisions incorporate important social insights crucial for cooperative societies.

This conclusion implies a need to revise the idea that emotion/intuition is the enemy of reason (Damasio 1994). It is not in dispute that these two systems may often be in conflict. Rather, the data suggest that decisions dictated by reason are not always good, while decisions dictated by emotion are not always bad. Damasio's key idea (the somatic marker hypothesis) is that patients with VMPC lesions make bad decisions because these decisions are no longer guided by emotions. In some circumstances, this lack of guidance by emotions can lead to better decisions (e.g. Shiv *et al*. 2005), but in most situations the lack of emotional guidance leads to bad decisions (Bechara & Damasio 2005). In economic decision making, we ignore our intuitions and emotions at our peril.
